# Microstructure Evolution and High-Temperature Dimensional Stability of Silica-Based Ceramic Cores via Modification of Alumina Nanopowder in Digital Light Processing 3D Printing

**DOI:** 10.3390/ma19071339

**Published:** 2026-03-27

**Authors:** Xin Li, Xin Chen, Yuan Si, Jie Wang, Chong He, Xiqing Xu, Shuxin Niu

**Affiliations:** 1 Science and Technology on Advanced High Temperature Structural Materials Laboratory, Beijing Institute of Aeronautical Materials, Beijing 100095, China; lx_ceramic@163.com (X.L.);; 2 School of Materials Science and Engineering, Chang’an University, Xi’an 710061, China; 3 School of Textile Science and Engineering, Wuhan Textile University, Wuhan 430200, China

**Keywords:** silica-based ceramic cores, 3D printing, dimensional stability, shrinkage, alumina nanopowder

## Abstract

3D printing represents a promising fabrication technology for silica-based ceramic cores, which are essential components in the casting of turbine blades, but it is faced with poor high-temperature dimensional stability. Herein, alumina nanopowder was utilized as a modifier agent in digital light processing (DLP) 3D printing of silica-based ceramic cores, and systematic investigations were conducted on the microstructure and properties of ceramic cores throughout sintering and casting dependent on the content of alumina nanopowder (0–1.0 wt.%). Alumina nanopowder increased the sintering barrier of fused silica, significantly reducing the shrinkage in sintering and simulated casting, while improving high-temperature dimensional stability. Even though the alumina nanopowder led to decreased room-temperature and high-temperature flexural strengths attributed to inhibited densification and crystallization, the strengths met investment casting requirements after PVA solution strengthening. Excessive alumina nanopowder (0.8–1.0 wt.%) resulted in poor interlayer bonding and particle spalling, unfavorable to the structural integrity in casting. The optimal alumina content was 0.6 wt.%, which balanced sintering shrinkage of 1.86%, shrinkage of 4.41% after simulated casting, room-temperature flexural strength of 11.13 MPa, high-temperature flexural strength of 31.29 MPa, high-temperature creep deformation of 0.55 mm, and surface roughness of 1.815 μm. This research proposes an effective strategy for the optimization of 3D-printed silica-based ceramic cores in the manufacture of complex hollow turbine blades.

## 1. Introduction

The aeroengine is the core component of an aircraft, and its thrust-to-weight ratio [1] is a key performance index. The higher thrust-to-weight ratio requires a higher inlet temperature at the front end of the turbine, and the turbine blades are simultaneously faced with high temperature, airflow impact and centrifugal force [2,3]. Casting superalloys such as high-temperature single crystal alloy are the preferred materials for turbine blades, but their service temperature has approached the limit [4]. In this condition, designing the air cooling structure [5] in blades becomes the optimal approach to reducing inlet temperature, enhancing the heat dissipation capacity of the blade, and upgrading the performance of the turbine blade.

The hollow blade with complex air-cooled structure has strict requirements on the material and structure, and it is mostly manufactured by investment casting. A ceramic core [6,7,8] is the key component to form the inner cavity, which needs to withstand multiple harsh working conditions, including resistance to liquid immersion and flushing during strengthening, resistance to high-temperature and low shrinkage during roasting and dewaxing, resistance to impact without cracking during wax injection, shape preservation without displacement during shell making, and resistance to alloy liquid impact with high-temperature strength during casting [9,10]. Among different ceramic cores, silica-based ceramic core [11,12,13] has good thermal stability, low coefficient of thermal expansion, easy control of shape and position accuracy, and has excellent leachability in alkaline solutions, which reduces the difficulty of blade cavity cleaning, improves the yield of castings, and meets the stringent requirements of investment casting for aeroengine blades.

With the rapid development of the aviation industry, the inner structure of turbine blades is becoming more and more complex. The traditional molding methods of ceramic core, such as hot injection and slip casting, are facing the industry problems of long cycle and high cost in design and manufacturing of complex molds [14,15]. In contrast, additive manufacturing [16,17,18], i.e., 3D printing, is an advanced manufacturing technology based on digital models, which can manufacture complex parts by accumulating materials layer by layer. Additive manufacturing technology can achieve rapid prototyping of complex ceramic core by slicing the digital model of the target part and forming the target part layer by layer with raw materials, without relying on molds. It benefits from the advantages, including short production cycle, high molding accuracy and flexible performance tuning [19,20]. For ceramic materials, digital light processing printing technology (DLP) [21,22,23] has become the mainstream method to prepare complex ceramic core owing to its advantages of high printing precision, superior surface finish, and rapid forming efficiency.

During directional solidification of superalloys, the silica-based ceramic cores are prone to severe casting shrinkage and high-temperature creep deformation [24,25], which directly undermines the dimensional accuracy of aeroengine blade inner cavities and compromises their overall forming quality. To ensure the dimensional accuracy and service performance of ceramic core and turbine blade, scholars have effectively improved the resistance of ceramic cores against casting shrinkage and creep deformation at high temperatures by means of particle grading [26] or appropriately increasing the sintering temperature [27]. However, these techniques significantly reduce the porosity of the core and adversely affect the core leaching process after casting. In our previous work [28], mullite whiskers were grown in situ in silica matrix to benefit the high-temperature accuracy; nevertheless, the introduction of impurity elements reduces the chemical compatibility between the core and the casting.

Alumina nanopowder [29,30] has high-temperature resistance, chemical stability and nanoscale high activity, which improves the high-temperature resistance to shrinkage and softening by modifying the silica matrix, while avoiding the introduction of impurities, and aligns with the molding and service requirements of ceramic cores manufactured by 3D printing. Accordingly, silica-based ceramic cores were fabricated via DLP 3D printing herein, and alumina nanopowder was employed as a modifier to explore the evolution of microstructure and phase composition in the silica-based ceramic core during sintering and casting, and to reveal the mechanism of alumina nanopowder on the high-temperature dimensional stability of the ceramic core.

## 2. Experimental Details

### 2.1. Preparation of Ceramic Slurries

In this paper, fused silica powder (purity >99.5%, D_50_ = 6 μm, Lianyungang Xinhai Quartz Products Co., Ltd., Lianyungang, China) was employed as the matrix powder, zirconium silicate (purity >99%, D_50_ = 20.51 μm, Andake (Jiangsu) Ceramics Co., Ltd., Changzhou, China) as the mineralizer to resist the high-temperature softening of fused silica during casting, and alumina nanopowder (purity >99%, D_50_ = 50 nm, Aladdin, Shanghai, China) was added in contents of 0–1.0 wt.% to replace the mixture of fused silica and zirconium silicate. The above ceramic powders were primarily mixed in a 3D motion mixer for 1 h, before which alumina nanopowder was treated via vacuum drying and ultrasonic dispersion to improve the dispersibility. The detailed composition of the ceramic powders was listed in Table 1.

The liquid phase of the slurry contained 45.5 wt.% of oligomers, 45.5 wt.% of monomers, 3 wt.% of photoinitiator, and 6 wt.% of dispersant. Polyurethane acrylate was used as the oligomer. The monomer is composed of HDDA, DPGDA, and TMPTA in the mass ratio of 6:3:1. BYK-111 was used as the dispersant, and the photo-polymerization reaction was initiated by TPO-L. The detailed composition of the photosensitive resin was based on a previous work [31]. The mixed ceramic powders along with the photosensitive resin were milled in a vertical planetary ball miller (DECO-PBM-V-4L, Deco-Mill, Changsha, China) at 600 rpm for 6 h, during which 6 wt.% of BYK-111 was employed as dispersant, to ensure the uniform distribution of powder in the system. Finally, a ceramic slurry with a solid loading of 60 vol% was obtained.

### 2.2. 3D Printing and Sintering

**Figure 1:**
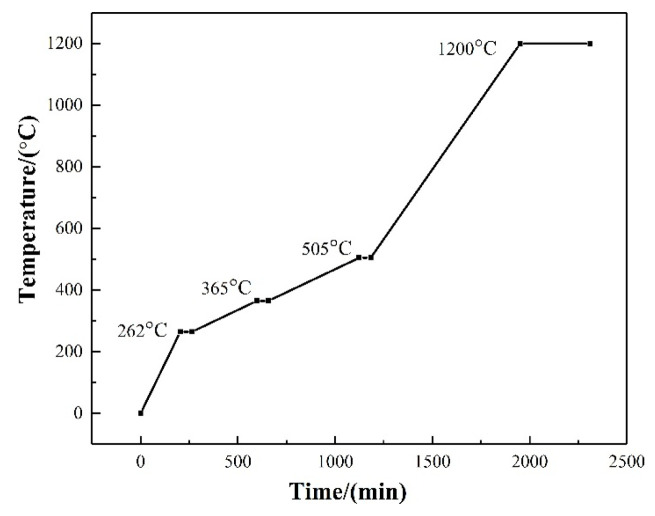
The program for debinding and sintering [32].

A DLP 3D printer, manufactured by Beijing Ten Dimensions Technology Co., Ltd. (Beijing, China), was adopted for the sample fabrication. The green bodies were printed using the ceramic slurry with curing power of 20 mW/cm^2^ for 3 s per layer, slice thickness of 100 μm. The thickness of the curing layer was evaluated using a spiral micrometer after exposure and removal of uncured photosensitive resin. The measurement was carried out taking average value on the center point and four corner positions of the square cured film. After printing, the as-printed green bodies were detached from the build platform and subjected to ultrasonic cleaning in ethanol for 10 min to remove residual slurry. The green samples were designed with a fixed dimension of 55 mm × 10 mm × 4 mm (length × width × height). Subsequent sintering was conducted at 1200 °C for 6 h, during which the ceramic cores were buried in calcined α-alumina powders, which is a commonly used filler in sintering of silica-based ceramic cores in industry due to its high-melting point, high-temperature chemical stability. The program was set to include a debinding and sintering stage, as shown in Figure 1, based on our previous work [32], during which the furnace temperature was held at 262 °C, 365 °C and 505 °C, respectively, for 1 h to eliminate the organic resin completely, and then rose to 1200 °C and maintained for 6 h, as the optimized temperature was confirmed in a previous work [7]. Finally, silica-based ceramic cores with different mass fractions of alumina nanopowder were successfully prepared. To verify the performance of ceramic cores in investment casting, simulated casting was carried out, where the as-sintered ceramic cores were heated to 1540 °C with a heating rate of 5 °C/min and isothermally held for 1 h.

### 2.3. Testing and Characterization

For the ceramic cores after sintering, Archimedes’ method was utilized for the bulk density, water absorption and apparent porosity, and mercury porosimetry (PoreMaster 60GT, Quantachrome, Boynton Beach, FL, USA) was adopted to analyze the pore size distribution. The viscosity of slurries were measured using a digital rotary viscometer (NDJ-8S, Shanghai Pingxuan Scientific Instrument Co., Ltd., Shanghai, China) with an accuracy of ±2% and a measurement range from 1 to 2 × 10^6^ mPa·s. A roughness tester (Marsurf PS1, Mahr, Göttingen, Germany) was conducted to assess the surface roughness on the surface vertical to the printing layer, and each test was based on the average value of 5 samples.

A S4800 SEM (Hitachi, Tokyo, Japan) was employed to detect the microstructural characteristics, and a D8 Advance XRD (Bruker, Karlsruhe, Germany) to manage the phase identification. A WDW-10 testing machine (Changchun Kexin Test Instrument, Changchun, China) was utilized to determine the flexural strength at room temperature and 1540 °C, with loading direction set parallel to the interlayer gaps, and each test was based on the average value of 5 samples. The high-temperature deflection was tested in terms of the double support cantilever beam method, using samples of 120 mm × 4 mm × 2 mm, whose interlayer gaps were parallel to the direction of gravity. The sample was placed on a ceramic bracket with a span of 100 mm, and heated in a high-temperature furnace at 1540 °C for 30 min. The hanging height of the cantilever sample was recorded as the high-temperature deflection, and each test was based on the average value of 5 samples.

To assess the service performance of the ceramic cores for high-temperature casting applications, a comprehensive heat treatment was implemented. Dimensional stability was evaluated via the linear shrinkage of the samples along three-dimensional directions after isothermal holding at 1540 °C for 1 h, and each test was based on the average value of 8 samples. In addition, simulation on the dimensional changes in ceramic cores during heating and cooling in the entire casting process was conducted with a DIL402 dilatometer (Netzsch, Selb, Germany) under air atmosphere, with the test temperature ranging from 25 to 1600 °C and a heating rate of 5 °C/min applied.

## 3. Results and Discussion

### 3.1. Phase Composition and Microstructure of Ceramic Cores After Sintering

**Figure 2:**
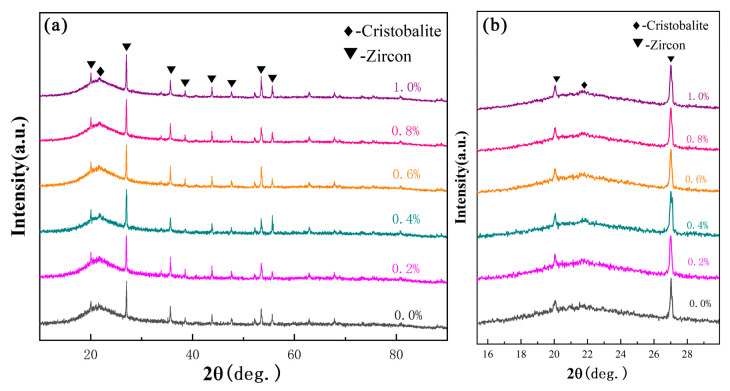
(**a**) XRD patterns and (**b**) enlarged image of ceramic cores with different contents of alumina nanopowder after sintering at 1200 °C.

Figure 2a showed the XRD patterns of ceramic cores with different alumina nanopowder contents after sintering. It is because that, limited by the detection resolution of XRD, the content of alumina nanopowder used in this work is lower than 1.0 wt.%, resulting in non-detection of alumina phase. Therefore, only the cristobalite phase and zircon phase were detected, without peaks for corundum phase, and the peaks for cristobalite are very weak as most of the silica is in an amorphous state. According to the normalized enlarged image in Figure 2b, the diffraction peak intensities of the cristobalite phase in all samples were basically the same, indicating that alumina nanopowder had little effect on the formation of cristobalite phase in the amorphous matrix after sintering.

**Figure 3:**
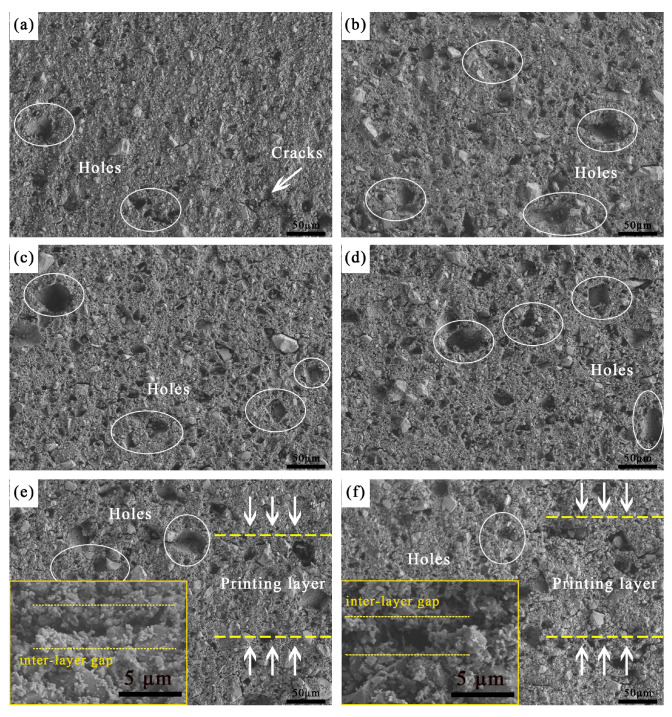
SEM on the surface of sintered ceramic core with different fractions of alumina nanopowder (**a**) 0.0 wt.%; (**b**) 0.2 wt.%; (**c**) 0.4 wt.%; (**d**) 0.6 wt.%; (**e**) 0.8 wt.%; (**f**) 1.0 wt.%.

Figure 3 showed the surface morphologies of ceramic cores with varying fractions of alumina nanopowder after sintering. The core surfaces were relatively flat, yet all exhibited varying degrees of particle spalling, a phenomenon that was more severe in the cores containing alumina nanopowder. There are three reasons for such particle spalling. Firstly, ceramic powders with large particles tend to deflect under ultraviolet light irradiation during printing, which may result in incomplete curing of large particles, while small pores remain in the green cores during printing [33]. Secondly, during debinding, the organic matter coating the ceramic particles volatilizes gradually at an elevated temperature, which may carry away some small-sized ceramic powders, thus leaving small pores on the core surfaces [6]. Finally, during sintering, the high refractoriness of alumina nanopowder increases the sintering barrier between fused silica particles, leading to a low degree of sintering and weak bonding strength between ceramic particles, which causes partial particle spalling during cleaning after furnace cooling. According to Figure 3, when the alumina nanopowder content was less than 0.8 wt.%, the printing layers and interlayer gaps were hardly observed on the surfaces. In contrast, with 0.8 wt.% and 1.0 wt.% alumina nanopowder, the interlayer gaps were detected, as marked in the enlarged view between yellow dashed lines, developing into gaps and weak interfaces due to incomplete bonding between adjacent printing layers. Based on the interlayer gaps, the printing layers were marked between the arrows, and the thickness was approximately 100 μm, matching the thickness of the printing layer in the experiment configuration. Similar interlayer structural characteristics have been documented in numerous studies [31,34], and the interlayer gaps can adversely affect the casting performance of ceramic cores.

**Figure 4:**
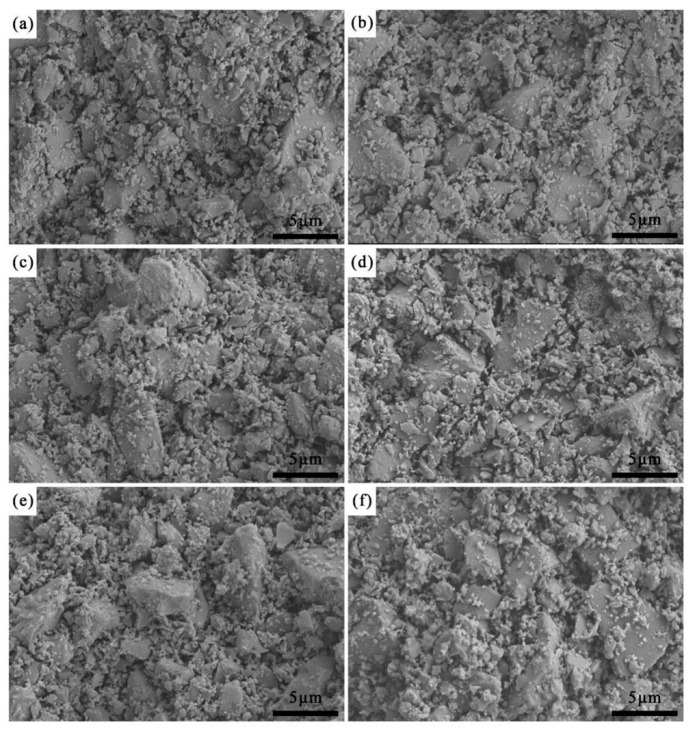
Fracture surface SEM images of sintered ceramic core with different alumina nanopowder contents: (**a**) 0.0 wt.%; (**b**) 0.2 wt.%; (**c**) 0.4 wt.%; (**d**) 0.6 wt.%; (**e**) 0.8 wt.%; (**f**) 1.0 wt.%.

Figure 4 presented the fracture SEM micrographs of ceramic cores with varying fractions of alumina nanopowder after sintering. It was shown that, the fracture morphologies showed little difference, and the fracture mode was dominated by intergranular fracture. The fracture surfaces revealed that large ceramic particles formed the skeleton, with small particles mostly distributed on the surfaces of large particles or in the voids, and the size of the largest ceramic particle skeleton was approximately 6 μm. In Figure 4a, the ceramic particles were relatively tightly bonded with small voids between them; with the increase in alumina nanopowder content, the bonding between particles became loose, and the small pores on the fracture surfaces gradually transformed into large pores.

**Figure 5:**
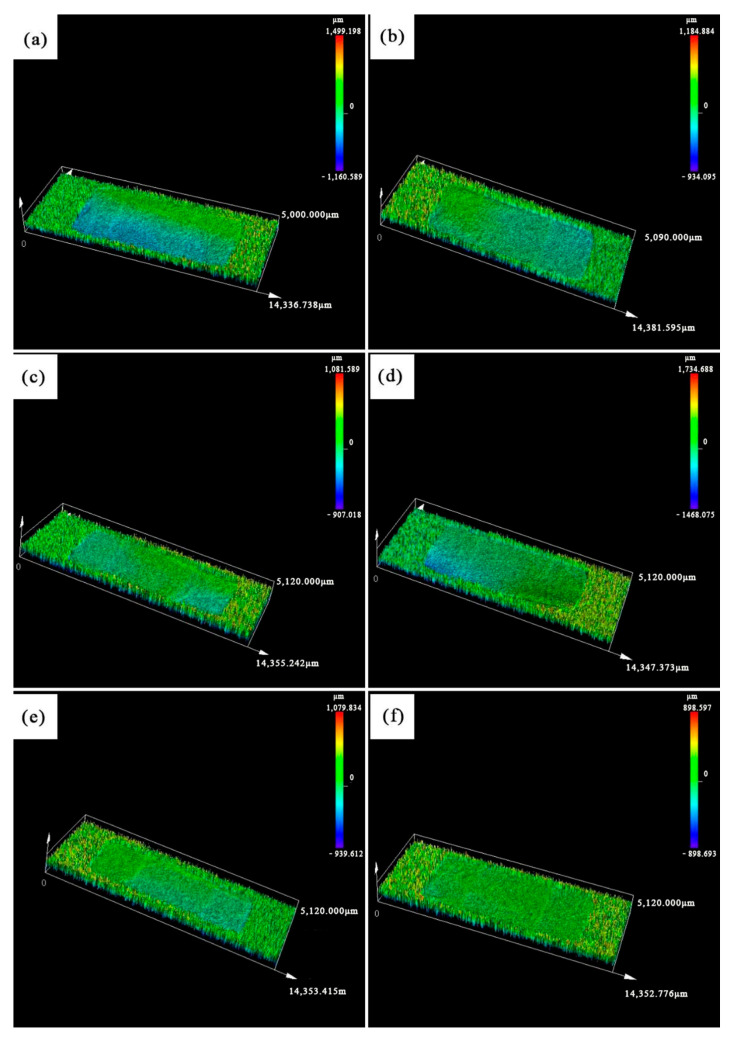
Three-dimensional scanning of fracture surface of sintered ceramic core with (**a**) 0.0 wt.%; (**b**) 0.2 wt.%; (**c**) 0.4 wt.%; (**d**) 0.6 wt.%; (**e**) 0.8 wt.%; (**f**) 1.0 wt.% of alumina nanopowder.

Figure 5 showed the 3D scanning images of the fractures of ceramic cores with different alumina nanopowder contents after primary sintering. The fracture morphology of the cores gradually became flatter after the introduction of alumina nanopowder, and the height of fracture protrusions and the roughness decreased with the increase in alumina nanopowder content. The fracture morphology was closely related to the bonding force between printed layers. In Figure 5a, due to the high sintering degree of the sample, the bonding strength between adjacent printed layers was high, resulting in a high flexural strength; therefore, the adhesion between adjacent layers during fracture led to a large height difference in the fracture morphology. In Figure 5b–f, with increasing fraction of alumina nanopowder, the sintering degree decreased, and the interlayer bonding strength became poorer with lower flexural strength. Under external load, the printed layers were prone to delamination, i.e., fracture occurred along the interlayers, which resulted in a flatter fracture morphology with a small height difference. In Figure 5, the fracture surface is parallel to the interlayer defects in Figure 3. For samples with low contents of alumina nanopowders (0–0.6 wt.%), the interlayer cracks are not obvious, suggesting excellent interlayer bonding and superior mechanical property. In this condition, the fracture intersects across different printing layers, leading to intersecting fracture surfaces, and the roughness of the fracture surface turns rough. However, interlayer cracks are severe in samples with high contents of alumina nanopowders (0.8–1.0 wt.%), suggesting weak interlayer bonding and poor mechanical property. In this condition, the fracture extends along the existing interlayer cracks easily, leading to single fracture surface with low roughness. Therefore, the high contents of alumina nanopowders lead to serious interlayer cracks, and further result in low roughness in fracture surface.

### 3.2. Properties of Ceramic Core After Sintering

**Figure 6:**
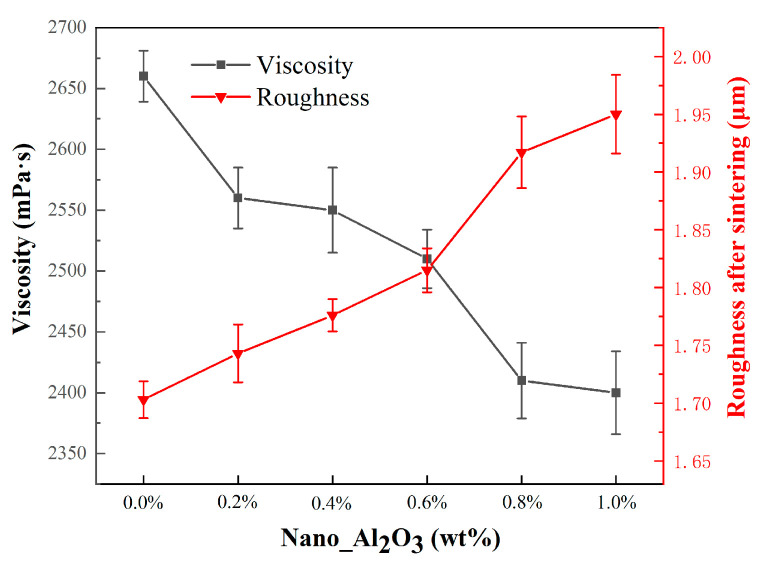
The effect of alumina nanopowder on the slurry viscosity and surface roughness of the sintered ceramic core.

Figure 6 illustrated the viscosity of ceramic slurries dependent on alumina nanopowder content. Overall, the slurry viscosity declines systematically with increasing percent of alumina powder. When the alumina nanopowder content was 0.2 wt.%, the slurry viscosity dropped sharply from 2660 mPa·s to 2560 mPa·s, after which the slurry viscosity declined gradually from 2550 to 2400 mPa·s when the percent of alumina powder rose from 0.2 wt.% to 1.0 wt.%. The viscosity of each ceramic slurry was less than 3000 mPa·s, meeting the viscosity requirements for 3D printing ceramic slurries, due to the extremely small particle size and high dispersibility of alumina nanopowder.

Figure 6 also presented the surface roughness of ceramic cores with varying percents of nano alumina sintered at 1200 °C. Overall, the surface roughness of the ceramic cores increases with the rise in alumina nanopowder content. The surface roughness increased slowly when the alumina nanopowder content was less than 0.6 wt.%; specifically, the surface roughness values of the ceramic cores were ranging from 1.703 μm to 1.815 μm at the alumina nanopowder contents from 0.0 wt.% to 0.6 wt.%, respectively. In contrast, the surface roughness rose sharply when the alumina nanopowder content exceeded 0.6 wt.%, reaching 1.917 μm and 1.950 μm with 0.8 wt.% and 1.0 wt.% alumina powders, respectively. After the green ceramic cores are sintered, the volatilization of organic matter at the interfaces between printing layers led to the formation of gaps or the spalling of surface particles. Therefore, the increased spacing between printed layers and the spalling of ceramic particles on the surface both contributed to the rougher surface, which will impair the surface quality of blade inner cavities. Even though the alumina nanopowder led to rougher surface, it well met the requirement in casting (within 3.2 μm).

**Figure 7:**
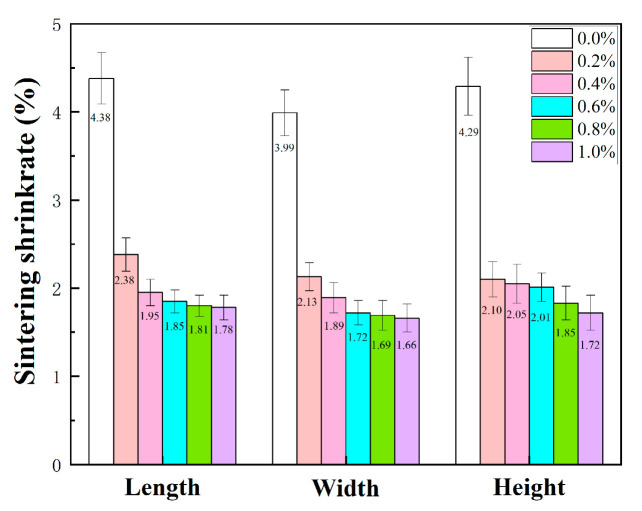
The effect of alumina nanopowder on the linear shrinkage of ceramic core during sintering at 1200 °C.

Figure 7 showed the three-dimensional shrinkage of samples with different fractions of alumina nanopowder after sintering at 1200 °C. It was suggested that, along the length, width and height axes, the sintering shrinkage of the ceramic cores dropped markedly as the alumina content rose, with largest shrinkage in length and smallest value in width. As the alumina content was elevated from 0.0 wt.% to 0.2 wt.%, the sintering shrinkage was significantly inhibited, and each of the three-dimensional shrinkages showed a decrease with high consistency for the ceramic cores. In the absence of alumina nanopowder, the linear shrinkage values in three-dimensional directions of the samples were 4.38%, 3.99% and 4.29%, respectively. For alumina nanopowder content of 0.2 wt.%, the three-dimensional shrinkages were 2.38%, 2.13% and 2.10%, respectively. The further doping in alumina from 0.2 wt.% to 1.0 wt.% led to gradual decline in linear shrinkage, with three-dimensional values of 1.78%, 1.66% and 1.72%, respectively. The shrinkage of DLP 3D-printed ceramics was mainly caused by interlayer shrinkage and sintering shrinkage between ceramic particles. After primary sintering, the more sufficient the sintering between adjacent layers and ceramic particles contributed to the higher the shrinkage. alumina nanopowder was uniformly distributed among ceramic particles, which increases the sintering barrier of fused silica and reduced the sintering driving force of fused silica powder [35], thus leading to a decrease in the average shrinkage from 4.22% to 1.72% for the sintered samples.

**Figure 8:**
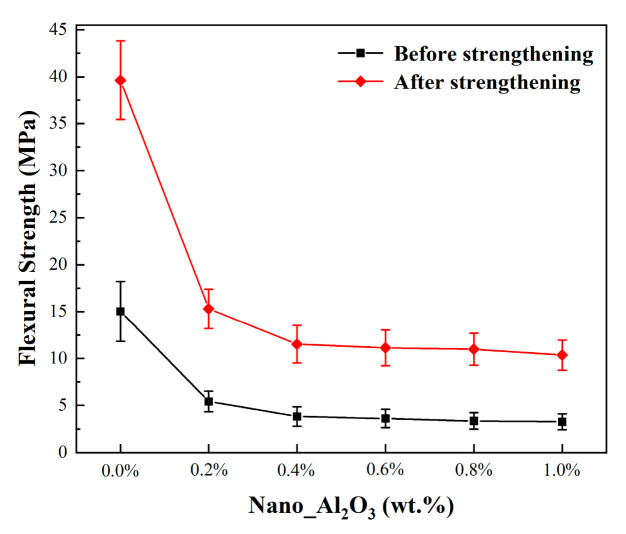
The influence of alumina nanopowder on the room-temperature flexural strength of sintered ceramic core before and after strengthening.

Figure 8 showed the flexural strength curves dependent on the alumina contents in the sintered samples. The room-temperature flexural strength of the samples decreased gradually with increasing fraction of alumina nanopowder. When the alumina nanopowder content increased from 0.0 wt.% to 0.2 wt.%, the room-temperature flexural strength of the ceramic cores dropped significantly from 15.02 MPa to 5.43 MPa, with a decrease of approximately 60%. As the alumina nanopowder content further increased to 1.0 wt.%, the strength further decreased and leveled off. At alumina nanopowder contents of 0.4–1.0 wt.%, the ceramic cores showed room-temperature flexural strength in the range of 3.83–3.27 MPa. The flexural strength at room temperature was closely related to the crystallization and densification during the sintering of ceramic cores. As a high-temperature refractory material, alumina nanopowder had a high refractoriness, which hindered the grain boundary migration and the grain growth inside the ceramic cores during sintering, contributing to a reduction in the strength of the samples with alumina nanopowder added.

In investment casting, room-temperature strength of above 8 MPa [14] is generally required for the ceramic cores, to ensure the structural integrity during wax mold pressing, while avoiding positioning deviation or fracture defects caused by low strength. Therefore, room-temperature strengthening was conducted on ceramic cores, which was immersed in PVA solution under vacuum for 1 h, followed by drying and curing at 120 °C for 2 h. The flexural strength after room-temperature strengthening was tested and plotted versus the alumina nanopowder content in Figure 8. For the ceramic cores with different alumina fractions, the flexural strengths at room temperature were ranging from 10.35 MPa to 39.63 MPa, which were about 3 times those before strengthening. Moreover, the flexural strengths higher than 10 MPa met the strength requirements of ceramic cores during the wax pressing.

**Figure 9:**
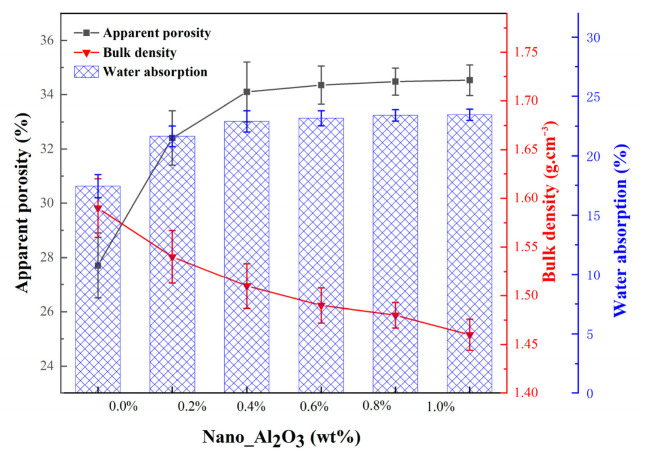
The effect of alumina nanopowder on apparent porosity, water absorption and bulk density of as-sintered samples.

Figure 9 illustrated the effects of alumina nanopowder content on the apparent porosity, water absorption and bulk density of ceramic cores. The doping of alumina nanopowder resulted in a marked rise in apparent porosity from 27.71% to 32.41%, a significant rise in water absorption from 17.43% to 21.64%, and a notable decline from 1.59 g/cm^3^ to 1.54 g/cm^3^ in bulk density. With the increase in alumina nanopowder content, the apparent porosity and water absorption showed a slight increasing trend, while the bulk density exhibited a slight decreasing trend. At an alumina nanopowder content of 1.0 wt.%, the ceramic core samples reached a maximum apparent porosity of 34.53%, a maximum water absorption of 23.46%, and a minimum bulk density of 1.46 g/cm^3^.

**Figure 10:**
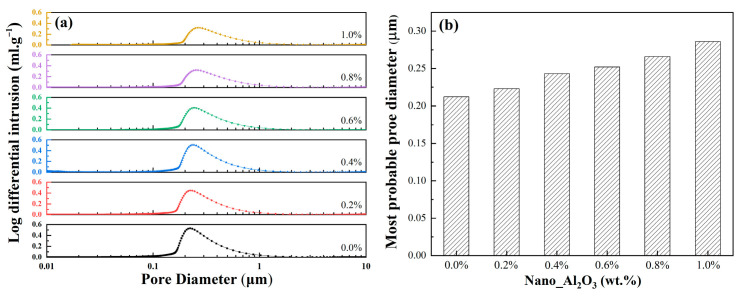
The effect of alumina nanopowder on the pore size of ceramic core after sintering (**a**) pore size distribution; (**b**) most probable pore diameter.

Figure 10a showed the distribution of pore size for as-sintered ceramic cores with different alumina contents. The pore sizes of the samples are mostly distributed ranging from 0.1 μm to 1 μm, and all the pore size distribution curves present a unimodal distribution, indicating a relatively concentrated pore size distribution of the samples. As the alumina nanopowder content increased, the pore size distribution curves shifted gradually to the right, suggesting increasing pore sizes. Figure 10b presented the most probable pore sizes of ceramic cores with different alumina nanopowder contents. The most probable pore size of the cores increases gradually with rising content of alumina nanopowder. With alumina nanopowder contents ranging from 0.0 to 1.0 wt.%, the most probable pore sizes of the cores were 0.212–0.286 μm. During sintering, the high refractoriness of alumina nanopowder increases the sintering barrier between fused silica particles, leading to a low degree of sintering and weak bonding strength between ceramic particles; therefore, the ceramic cores show increasing porosity in quantity and size with increasing alumina contents. In the leaching process of ceramic core, the alkaline solution can penetrate into the interior of the cores more easily through the pores, which accelerates the leaching efficiency of the ceramic cores after casting and thus improves the core removal inside the alloy blades.

### 3.3. Phase Composition and Microstructure of Ceramic Cores After Simulated Casting at 1540 °C

**Figure 11:**
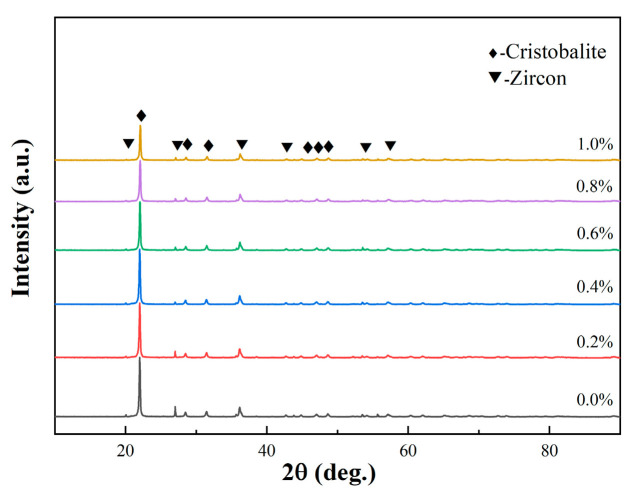
XRD patterns of ceramic cores with varying fractions of alumina nanopowder after heat treatment at 1540 °C.

Figure 11 showed the XRD patterns of ceramic cores with varying fractions of alumina powder after simulated casting at 1540 °C. With rising alumina content, the intensity of the diffraction peaks for cristobalite phase gradually weakened, while the peak intensity of the zircon remained basically unchanged. It was indicated that, alumina nanopowder had significant inhibitory effects on the crystallization of silica in simulated casting. By comparing with the XRD patterns in Figure 2, it was concluded that alumina nanopowder increased the crystallization temperature of fused silica, and thus inhibited the sintering of the cores at relatively low temperatures. According to previous studies [36], Al^3+^ ions were characterized by high charge and captured oxygen atoms easily, which resulted in the formation of oxygen atom vacancies in fused silica. This behavior made it difficult for oxygen atoms in fused silica to further order, thus restricting the formation of cristobalite phase based on the crystallization of amorphous silica.

**Figure 12:**
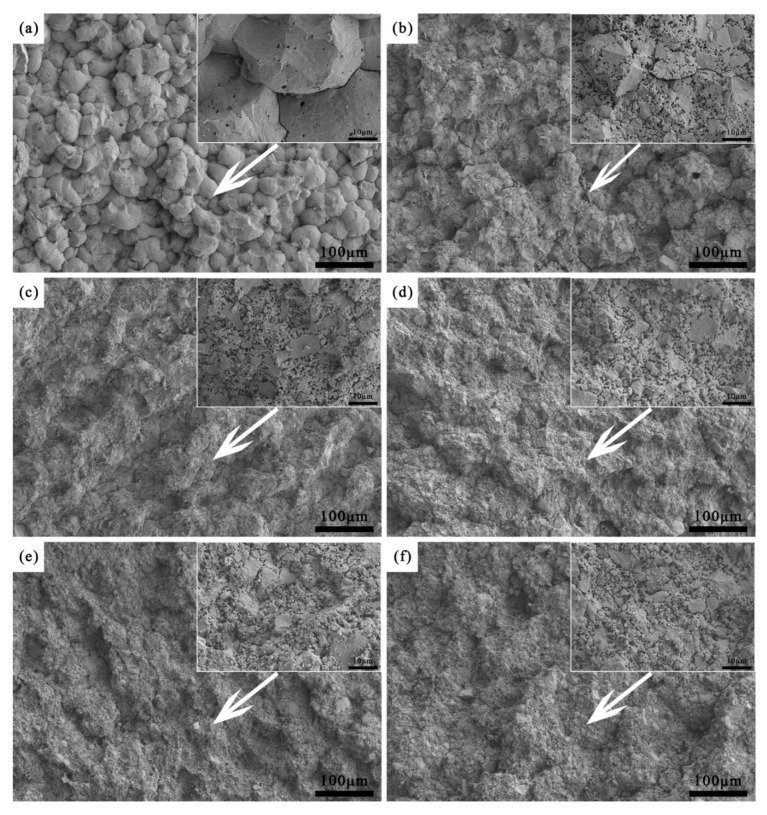
SEM taken from the fracture surface of as-casted ceramic core with different alumina nanopowder contents: (**a**) 0.0 wt.%; (**b**) 0.2 wt.%; (**c**) 0.4 wt.%; (**d**) 0.6 wt.%; (**e**) 0.8 wt.%; (**f**) 1.0 wt.%.

Figure 12 displayed the fracture surface microstructures of ceramic cores with varying fractions of alumina nanopowder after simulated casting at 1540 °C, with the high-magnification morphologies of each sample presented in the upper right corner of the images. Compared with the fracture morphologies after sintering at 1200 °C (Figure 4), the fracture densities of all samples were improved and the porosities were reduced after simulated casting. In Figure 12a, large sintering necks were formed between ceramic particles after further densification in simulated casting. Under high temperature, fine ceramic particles grew rapidly through grain boundary migration, forming coarse ceramic particles with diameters ranging from 50 to 100 μm. The density of the overall fracture surface was high, the bonding strength between particles was strong, along with large numbers of cracks. Such cracks were caused by volume shrinkage accompanying the polymorphic phase transition of cristobalite. After the doping of 0.2 wt.% alumina nanopowder (Figure 12b), the degree of densification between the adjacent particles decreased rapidly, the fracture morphology appeared loose, with higher porosity and fewer cracks; the bonding strength between particles were weakened, and the skeletons of approximately 50 μm were clearly visible. As the alumina nanopowder content further increased (Figure 12c–f), the fracture morphology became looser with larger quantities of pores and smaller skeleton size.

### 3.4. Properties of Ceramic Cores After Simulated Casting

**Figure 13:**
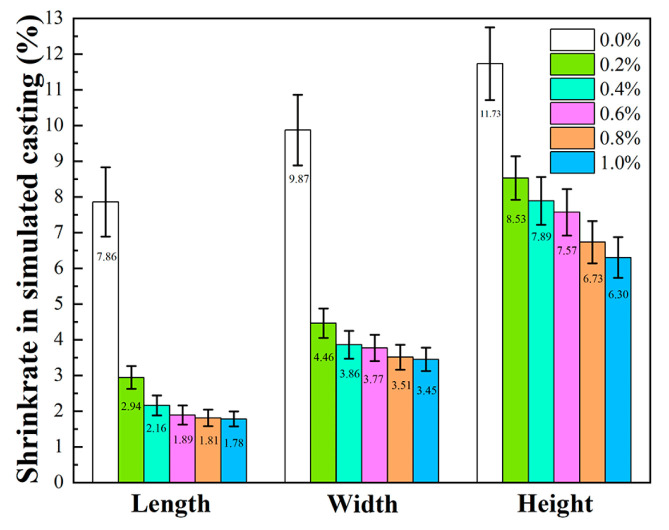
Effect of alumina nanopowder on shrinkage of ceramic cores after simulated casting at 1540 °C.

Figure 13 illustrated the shrinkage of core samples after simulated casting at 1540 °C dependent on varying fractions of alumina nanopowder, with highest shrinkage in height and lowest in length during simulated casting. After the introduction of alumina nanopowder, the shrinkage decreased significantly in simulated casting. At an alumina nanopowder content of 0.2 wt.%, the average value of three-dimensional shrinkage in simulated casting dropped from 9.82% to 5.31%. With the increase in fraction of alumina nanopowder, the shrinkage in simulated casting of the samples declined slowly. With 1.0 wt.% alumina nanopowder, the average value of three-dimensional shrinkages in simulated casting were 3.84%. In agreement with the behavior in Figure 12, the addition of alumina nanopowder inhibited the further densification between ceramic particles in the ceramic cores, thereby reducing the shrinkage during simulated casting.

**Figure 14:**
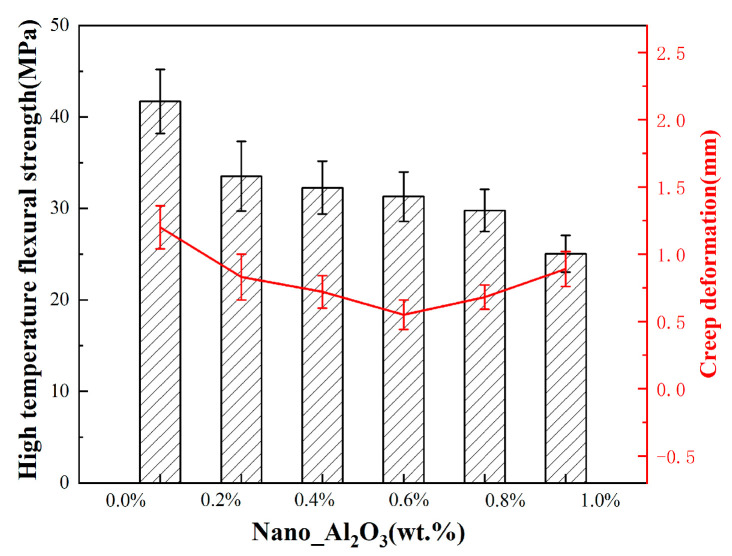
High-temperature strength and creep deformation of ceramic cores with different alumina nanopowder content.

The high-temperature strengths of the samples with different fractions of alumina nanopowder were evaluated, as illustrated in Figure 14. The ceramic cores showed a decreasing trend in high-temperature strength with the rise in alumina content. The core without alumina nanopowder attained a maximum strength of 41.70 MPa at high temperature, while the value reached a minimum of 25.05 MPa with 1.0 wt.% alumina nanopowder, which was still higher than the requirement of high-temperature strength (15 MPa) [14] for investment casting. According to the XRD patterns in Figure 11, the reduction in cristobalite precipitation, namely the inhibition of devitrification, led to an increase in the viscous fluidity of silica-based ceramic cores in high-temperature casting, and ultimately resulted in the degraded strength of ceramic cores at high temperature. Figure 14 also showed the high-temperature deflection of different ceramic cores at 1540 °C. As the content of nano-alumina increased from 0 wt.% to 0.6 wt.%, the high-temperature deflection of the ceramic core decreases from 1.2 mm to 0.55 mm. However, as the alumina content further increased to 1.0 wt.%, the high-temperature creep resistance of the ceramic core is significantly weakened, resulting in an increase in high-temperature deflection to 0.89 mm. On the one hand, the alumina hinders the particle rearrangement of silica particles in the ceramic core during secondary sintering, leading to an improvement in shape stability. On the other hand, the addition of alumina reduces the high-temperature strength of the ceramic core, thus weakening its ability to resist high-temperature deformation. Therefore, the high-temperature deflection of the ceramic core first decreased and then increased with increasing alumina content, and the minimum value was 0.55 mm with alumina content of 0.6 wt.%.

**Figure 15:**
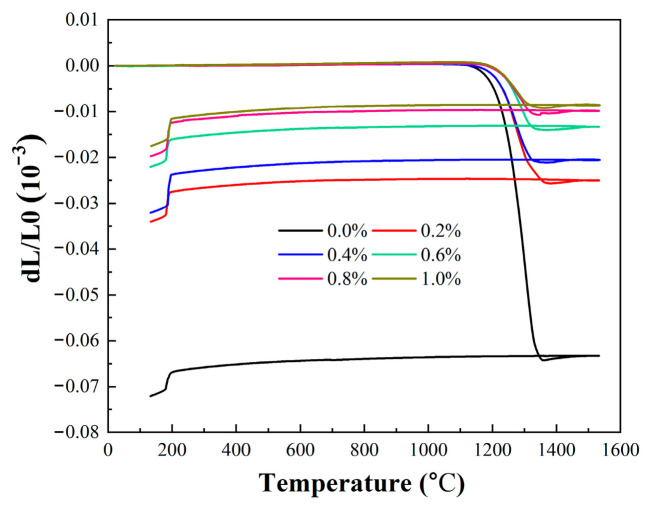
The dimensional changes in ceramic cores with different contents of alumina nanopowder during heating and cooling in the entire casting process.

Figure 15 exhibited the thermal expansion curves of samples with different fractions of alumina nanopowder to reveal the dimensional changes in ceramic cores during heating and cooling in the entire casting process. All curves showed the same trend at the initial heating stage, with almost no change observed in the thermal expansion curves. Notably, there was no inflection point in any of the curves at around 200 °C, indicating low content of cristobalite content in each sample without phase transformation of cristobalite, which was consistent with the XRD patterns in Figure 2. As the temperature was elevated to 1100–1200 °C, the samples started to soften, resulting in the inflection points in all curves. For ceramic cores with a alumina nanopowder content of less than 0.6 wt.%, the inflection points appear earlier with an obvious shrinkage phenomenon, due to the lower sintering temperature of ceramic cores with lower content of alumina nanopowder, leading to easier densification and shrinkage of the cores. In contrast, the inflection points appeared later for ceramic cores with alumina nanopowder content above 0.6 wt.%, which is associated with alumina nanopowder hindering the further densification of fused silica to a higher temperature, resulting in later formation of liquid phase. As the temperature further rose to 1300 °C, the cores begun to expand, and the linear thermal expansion rate increased gradually until 1540 °C, which was attributed to the phase transformation of cristobalite. In the beginning of the cooling stage, the linear shrinkage of the ceramic cores increases gradually with decreasing temperature. When the temperature dropped to approximately 200 °C, the α phase of cristobalite transformed into β-cristobalite [37], which consequently induced the formation of an inflection point in the curve and an elevated shrinkage degree of the samples. Ultimately, with increasing content of alumina nanopowder, the total linear shrinkage of the ceramic cores was markedly hindered, which was in agreement with those presented in Figure 13.

## 4. Conclusions

To improve the dimensional stability of DLP 3D-printed silica-based ceramic cores in high-temperature casting, alumina nanopowder was employed as a modifier, with main conclusions as follows:

1. The alumina nanopowder increased the sintering barrier of fused silica and hindered the densification of the sample. Therefore, the linear shrinkage of the cores in sintering and casting were highly reduced, and the high-temperature dimensional stability was improved. However, 0.8–1.0 wt.% alumina nanopowder resulted in poor interlayer bonding with obvious delamination or particle spalling, which destroyed the structural integrity during casting.
2. Alumina nanopowder led to degradation in the flexural strengths of the ceramic core both at room and high temperatures, due to the inhibition on the densification and crystallization. However, after strengthening with PVA solution, the flexural strength met the requirements for investment casting.
3. The alumina nanopowder content of 0.6 wt.% achieved a balanced performance, which effectively suppressed shrinkage from 4.42% to 1.86% in sintering and shrinkage from 9.82% to 4.41% in simulated casting. Furthermore, the sample with 0.6 wt.% alumina nanopowder showed flexural strength of 11.13 MPa at room temperature and 31.29 MPa at high temperature. Furthermore, the ceramic cores exhibited low roughness of 1.815 μm, a most probable pore size of 0.26 μm, which balances the surface quality and leaching efficiency. This work provides an effective method to superior dimensional stability of 3D-printed silica-based ceramic cores in casting.

## Figures and Tables

**Table 1:** Detailed composition of the ceramic powders in different samples.

Sample	Mass Percent of the Powders (wt.%)		
	Fused Silica Powder	Zirconium Silicate	Alumina Nanopowder
A-00	95	5	0
A-02	94.81	4.99	0.2
A-04	94.62	4.98	0.4
A-06	94.43	4.97	0.6
A-08	94.24	4.96	0.8
A-10	94.05	4.95	1

## Data Availability

The original contributions presented in this study are included in the article. Further inquiries can be directed to the corresponding authors.
